# Clinical study on postoperative inflammation and cardiac function improvement following valve replacement surgery

**DOI:** 10.3389/fcvm.2025.1584417

**Published:** 2025-11-27

**Authors:** Xuelian Li, Maosen Xu, Chen He, Shengwu Chao, Rong Zhou

**Affiliations:** 1 Department of Cardiology, Yancheng No.1 People’s Hospital, Yancheng, Jiangsu, China; 2 Zhongshan Hospital, Fudan University, Shanghai, China; 3 Navy 905 Hospital, Shanghai, China

**Keywords:** valve replacement surgery, postoperative inflammation, C-reactive protein, cardiac function recovery, risk factors

## Abstract

**Objective:**

To investigate the impact of C-reactive protein (CRP) levels measured on the third day after valve replacement surgery on cardiac function recovery at 3 months postoperatively and to analyze its association with other clinical risk factors, thereby providing reference data for perioperative management.

**Methods:**

A retrospective analysis was conducted on 188 patients who underwent valve replacement surgery. Based on predefined inclusion and exclusion criteria, using a postoperative CRP level of 150 mg/L as the grouping standard. Patients were divided into a high-inflammation group (100 cases) and a low-inflammation group (88 cases) based on CRP levels on postoperative day 3. Baseline characteristics, preoperative BNP levels, LVEF, intraoperative parameters, postoperative inflammation levels, and complications were compared between the two groups. Postoperative LVEF, BNP levels at 1 week and 3 months, and total hospital stay were evaluated. Univariate and multivariate logistic regression analyses were used to identify factors influencing cardiac function improvement at 3 months in the high inflammation group. Data were analyzed using SPSS, with a *P*-value <0.05 considered statistically significant.

**Results:**

The high inflammation group showed a lower cardiac function improvement rate at 3 months (71% vs. 85.2%, *P* = 0.019) and a longer hospital stay (19 [17, 21] vs. 18 [16, 20], *P* = 0.013). LVEF at 1 week and 3 months postoperatively was lower (43.55 ± 4.45 vs. 46.31 ± 4.84, *P* < 0.001; 50.96 ± 11.25 vs. 54.82 ± 6.52, *P* = 0.004), while BNP levels were higher (751.63 ± 52.12 vs. 632.90 ± 305.10, *P* = 0.001; 289.56 ± 108.51 vs. 186.99 ± 84.20, *P* < 0.001). Univariate analysis showed that age, hypertension, diabetes, preoperative BNP levels, preoperative LVEF, and postoperative complications were associated with cardiac function improvement (*P* < 0.05). Multivariate analysis identified age (OR = 5.83, 95% CI 1.79–19.01, *P* = 0.003) and lower preoperative LVEF (OR = 5.96, 95% CI 1.47–24.12, *P* = 0.012) as independent risk factors for poor cardiac function recovery.

**Conclusion:**

Elevated CRP levels on the third day after valve replacement surgery significantly impact cardiac function recovery and prolong hospital stay. Moreover, advanced age and lower preoperative LVEF are independent risk factors for poor cardiac function recovery.

## Introduction

Valvular heart disease refers to the abnormal structure or function of the four major valves of the heart (1). The aortic valve is located between the left ventricle and the aorta. Its common causes include congenital malformation, thickening and adhesion of the valve caused by rheumatic heart disease, and calcification caused by degeneration; The mitral valve is located between the left atrium and the left ventricle, which is susceptible to rheumatic fever caused by streptococcal infection, resulting in valve stenosis or insufficiency, and may also cause problems due to papillary muscle dysfunction caused by myocardial ischemia. Reflux is mostly induced by pulmonary hypertension, right ventricular enlargement and other factors; pulmonary valve connects right ventricle and pulmonary artery, which can cause pathological changes due to congenital dysplasia, infective endocarditis and so on. These different causes lead to structural or functional abnormalities of valves, and then lead to valvular heart disease, which has become an important inducement of cardiac dysfunction and heart failure, and seriously threatens the health of patients (2, 3).

The global prevalence of valvular disease is increasing with population aging, particularly affecting elderly patients with aortic stenosis and mitral regurgitation (4–6).

In addition to the traditional open chest valve replacement, transcatheter aortic valve implantation, as a new minimally invasive alternative, has attracted more and more attention because of its advantages of small trauma and quick recovery. The prostheses used in valve replacement are mainly divided into mechanical valve and biological valve, including xeno and homo. Source materials, each of which has different characteristics and applicable scenarios (6, 7). Valve replacement remains the most effective treatment for severe valve disease, especially in patients with irreparable or severe functional impairment. By accurately replacing damaged valves, the operation can significantly improve the hemodynamic status of the heart and effectively prolong the survival time of patients. However, even if the operation successfully restores the mechanical function of the valve, the postoperative inflammatory response and its long-term impact on cardiac structure and function are still key issues that need close attention in clinical practice (7, 8).

Studies have shown a close association between systemic inflammatory responses and cardiac dysfunction. C-reactive protein (CRP), a key marker of inflammation, is widely used to assess postoperative inflammatory levels and cardiac recovery (9, 10).

Furthermore, research suggests that the inflammatory response not only directly affects cardiac function but may also lead to postoperative cognitive dysfunction by disrupting the blood-brain barrier and triggering central nervous system inflammation (11, 12). Thus, it is necessary to investigate the pathological role of CRP in patients undergoing valve replacement surgery. Elevated CRP levels have been shown to contribute to myocardial fibrosis, cardiac remodeling, and impaired pump function.

Under surgical trauma and stress from cardiopulmonary bypass, inflammatory responses may worsen cardiac outcomes (13). However, some studies have explored the relationship between preoperative or early postoperative inflammation and cardiac function, limited research investigates the link between inflammatory status on postoperative day 3 and long-term cardiac recovery. Therefore, this study aims to bridge this gap by systematically evaluating the relationship between postoperative day 3 inflammation levels and long-term cardiac function recovery. The findings are expected to provide new clinical evidence for postoperative cardiac management and offer guidance for optimizing personalized treatment strategies.

## Materials and methods

### Patient selection

We conducted a retrospective analysis of patients who underwent valve replacement surgery at our hospital between June 2021 and August 2023. A total of 188 patients were enrolled based on predefined inclusion and exclusion criteria, using a postoperative CRP level of 150 mg/L as the grouping standard. Of these, 100 patients were assigned to the high-inflammation group, and 88 to the low-inflammation group. Postoperative follow-up lasted for 3 months, during which relevant data were collected via outpatient visits or telephone interviews.

### Inclusion criteria

1. Patients diagnosed with valvular disease by a cardiovascular specialist.
2. Patients who underwent open-chest valve replacement surgery.
3. Patients aged between 18 and 80 years, without severe systemic comorbidities or surgical contraindications.
4. Patients with complete medical records, including preoperative diagnosis, intraoperative details, and postoperative follow-up data.

### Exclusion criteria

1. Patients who underwent concurrent cardiac surgeries, such as cardiac tumor resection, coronary artery bypass grafting, or vascular surgery.
2. Patients with a history of valve-related surgery or those who had undergone ≥2 valve surgeries during the same hospitalization.
3. Patients with severe infections or immune system disorders (Rheumatic Heart Disease).
4. Patients who underwent valve replacement without cardiopulmonary bypass (CPB).
5. Patients with a follow-up period of less than 3 months or who were lost to follow-up.

All patients met the same inclusion and exclusion criteria. This study was reviewed and approved by the Ethics Committee of Yancheng No.1 People's Hospital. This study is in concordance with the declaration of Helsinki.

### Data collection

The preoperative variables in this study included general patient information (age, gender, and medical history such as hypertension and diabetes) and preoperative laboratory test results, including white blood cell count, red blood cell count, platelet count, coagulation parameters, brain natriuretic peptide (BNP), alanine transaminase (ALT), aspartate transaminase (AST), creatinine (Cr), and blood urea nitrogen.

Preoperative imaging assessments included echocardiographic measurement of left ventricular ejection fraction (LVEF). All preoperative tests were conducted within 3 days before surgery. If multiple tests were performed, the result closest to the surgery date was used. Perioperative parameters mainly included the duration of surgery and cardiopulmonary bypass (CPB) time. Postoperative data included inflammatory markers (CRP levels on postoperative days 1 and 3), cardiac function indicators (LVEF and BNP changes at 1 week and 3 months postoperatively), major organ complications, and total hospital stay.

### Postoperative management and evaluation indicators

All patients received standardized treatment based on the 2017 ACC/AHA Guideline Update for the Management of Patients with Valvular Heart Disease (14). The evaluation indicators included cardiac function improvement at 3 months, postoperative changes in LVEF and BNP levels, major organ complications, and total hospital stay. The criteria for evaluating cardiac function improvement were defined as follows (15, 16): an increase in LVEF of ≥10% compared to the preoperative value or a reduction in BNP of ≥30%, or an increase in LVEF of ≥5% accompanied by a BNP reduction of ≥20% was considered indicative of cardiac function improvement.

Conversely, patients not meeting these criteria were classified as having no improvement.

### Statistical analysis

All statistical analyses were performed using SPSS version 25.0 (IBM Corp., Armonk, NY, USA). Continuous variables with normal distribution were expressed as mean ± standard deviation, and comparisons between groups were conducted using the independent-sample *t*-test. Non-normally distributed continuous variables were presented as median (interquartile range) and compared using the Mann–Whitney *U* test. Categorical variables were presented as frequencies and percentages, and group differences were evaluated using the *χ*^2^ test. Univariate analysis was conducted to identify potential factors influencing postoperative inflammation and cardiac function improvement after valve replacement surgery. Variables with *P* < 0.05 in univariate analysis were included in multivariate logistic regression to identify independent factors associated with cardiac function improvement. All tests were two-sided, and a *P*-value <0.05 was considered statistically significant.

## Results

### Baseline characteristics

A total of 188 patients who underwent cardiac valve replacement surgery were included in the study. The high-inflammation group consisted of 100 patients with a mean age of 63.30 ± 10.24 years, including 58 males (58.00%) and 42 females (42.00%).

The low-inflammation group consisted of 88 patients with a mean age of 63.27 ± 9.38 years, including 54 males (61.36%) and 34 females (38.64%). No statistically significant differences were observed between the two groups in demographic characteristics, comorbidities, or preoperative laboratory tests (*P* > 0.05). Regarding cardiac function indicators, both groups showed no significant differences in left ventricular ejection fraction (LVEF) or B-type natriuretic peptide (BNP) levels (*P* > 0.05). Overall, the two groups were well-balanced in terms of preoperative baseline characteristics, providing reliable comparability for the subsequent analysis of postoperative outcomes and prognosis (Table 1).

**Table 1:** Comparison of preoperative clinical data between the two groups.

Variable	High inflammation group (100 cases)	Low inflammation group (88 cases)	Statistical value	*P*-value
Age (years)	63.30 ± 10.24	63.27 ± 9.38	*t* = 0.019	0.985
Gender				
Male	58 (58.00%)	54 (61.36%)	*Z* = 0.220	0.639
Female	42 (42.00%)	34 (38.64%)		
Diabetes	13 (13.00%)	9 (10.23%)	*Z* = 0.348	0.555
Hypertension	23 (23.00%)	20 (22.73%)	*Z* = 0.002	0.965
CRP (×10^12^)	3.84 ± 1.20	3.65 ± 1.01	*t* = 1.216	0.226
RBC (×10^9^)	4.86 ± 0.66	4.88 ± 0.58	*t* = 0.254	0.800
WBC (×10^9^)	7. 18 ± 1.51	7.42 ± 1.45	*t* = 1.120	0.264
Platelets (×10^9^)	220.84 ± 63.60	219.84 ± 55.51	*t* = 0.114	0.909
TT (s)	17.54 ± 1.16	17.86 ± 1.24	*t* = 1.858	0.065
APTT (s)	26. 16 ± 2.42	26.03 ± 2.29	*t* = 0.379	0.705
PT (s)	11.23 ± 0.75	11.25 ± 0.89	*t* = 0.140	0.889
FIG (g/L)	2.82 ± 0.65	2.85 ± 0.77	*t* = 0.261	0.794
AST (U/L)	25. 18 ± 7.50	25.46 ± 9.20	*t* = 0.233	0.816
ALT (U/L)	22.82 ± 8.78	23. 14 ± 9.38	*t* = 0.243	0.809
BUN (mmol/L)	4.71 ± 1.37	4.96 ± 1.61	*t* = 1.188	0.236
Cr (µmol/L)	62.09 ± 15.28	61.52 ± 16.39	*t* = 0.248	0.804
LVEF	36.02 ± 4.99	35.32 ± 4.39	*t* = 1.018	0.310
BNP (pg/mL)	1,362.92 ± 415.95	1,367.51 ± 357.59	*t* = 0.081	0.936

CRP, C-reactive protein; RBC, red blood cell count; WBC, white blood cell count; TT, thrombin time; APTT, activated partial thromboplastin time; PT, prothrombin time; FIG, fibrinogen; AST, aspartate transaminase; ALT, alanine transaminase; BUN, blood urea nitrogen; Cr, creatinine; LVEF, Left ventricular ejection fraction; BNP, B-type natriuretic peptide.

### Comparison of intraoperative and postoperative variables

There was no statistically significant difference between the high-inflammation group and the low-inflammation group in terms of surgery duration (278.07 ± 44.41 vs. 272.77 ± 43.38 min, *P* = 0.410) or cardiopulmonary bypass (CPB) time (127.70 ± 18.23 vs. 124.89 ± 17.81 min, *P* = 0.287). Similarly, CRP levels on postoperative day 1 did not differ significantly between the two groups (61.54 ± 18.12 vs. 61.48 ± 18.83, *P* = 0.981). However, cardiac function indicators revealed poorer postoperative recovery in the high-inflammation group. LVEF at 1 week and 3 months postoperatively was significantly lower in the high-inflammation group compared to the low-inflammation group (43.55 ± 4.45 vs. 46.31 ± 4.84, *P* < 0.001; 50.96 ± 11.25 vs. 54.82 ± 6.52, *P* = 0.004). Additionally, BNP levels at 1 week and 3 months postoperatively were significantly higher in the high-inflammation group (751.63 ± 52.12 vs. 632.90 ± 305.10, *P* = 0.001; 289.56 ± 108.51 vs. 186.99 ± 84.20, *P* < 0.001), suggesting a greater cardiac burden. No significant differences were observed in postoperative complications between the two groups, including pulmonary infections [16 (16.00%) vs. 10 (11.36%), *P* = 0.358], renal dysfunction [10 (10.00%) vs. 8 (9.09%), *P* = 0.833], or arrhythmias [14 (14.00%) vs. 13 (14.77%), *P* = 0.880].

In terms of hospitalization duration, the total hospital stay was significantly longer in the high-inflammation group compared to the low-inflammation group (19.00 [17.00, 21.00] vs. 18.00 [16.00, 20.00], *P* = 0.013) (Table 2), suggesting that elevated inflammation may delay recovery and prolong hospitalization. Finally, the proportion of patients with improved cardiac function was significantly lower in the high-inflammation group compared to the low-inflammation group [75 (75.00%) vs. 71 (80.68%), *P* = 0.019], with statistically significant differences (Table 3).

**Table 2:** Comparison of intraoperative and postoperative clinical data and total hospital stay between the two groups.

Variable	High inflammation group (100 cases)	Low inflammation group (88 cases)	Statistical value	*P*-value
Surgery duration (min)	278.07 ± 44.41	272.77 ± 43.38	0.825	0.410
CPB (min)	127.70 ± 18.23	124.89 ± 17.81	1.067	0.287
CRP (1 day) (mg/L)	61.54 ± 18.12	61.48 ± 18.83	0.023	0.981
LVEF (1 week) (%)	43.55 ± 4.45	46.31 ± 4.84	4.072	**<0.001**
LVEF (3 months) (%)	50.96 ± 11.25	54.82 ± 6.52	2.917	**0.004**
BNP (1 week) (pg/mL)	751.63 ± 52.12	632.90 ± 305.10	3.605	**0.001**
BNP (3 months) (pg/mL)	289.56 ± 108.51	186.99 ± 84.20	7.284	**<0** **.** **001**
Pulmonary infection	16 (16.00%)	10 (11.36%)	0.844	0.358
Renal dysfunction	10 (10.00%)	8 (9.09%)	0.045	0.833
Arrhythmia	14 (14.00%)	13 (14.77%)	0.023	0.880
Total hospital stay (days)	19.00 (17.00, 21.00)	18.00 (16.00, 20.00)	6.118	**0** **.** **013**

Bold indicates that a *P*-value less than 0.05 indicates a statistically significant difference in the experimental results.

CPB, cardiopulmonary bypass; CRP, C-reactive protein; LVEF: left ventricular ejection fraction; BNP, B-type natriuretic peptide.

**Table 3:** Comparison of cardiac function improvement outcomes at 3 months postoperatively between the two groups.

Outcome	High inflammation group (100 cases)	Low inflammation group (88 cases)	Statistical value	*P*-value
Improvement	75 (75.00%)	71 (80.68%)	5.461	**0.019**
No improvement	13 (13.00%)	29 (32.95%)		

Improvement: an increase in LVEF of ≥10% compared to the preoperative value or a reduction in BNP of ≥30%, or an increase in LVEF of ≥5% accompanied by a BNP reduction of ≥20% was considered indicative of cardiac function improvement. Conversely, patients not meeting these criteria were classified as having no improvement.

Bold indicates that a *P*-value less than 0.05 indicates a statistically significant difference in the experimental results.

### Analysis of prognostic risk factors for cardiac function in the high-inflammation group

According to the classification criteria, 100 patients with elevated CRP levels on the third postoperative day were included in this study. Sixteen potential factors influencing cardiac function improvement at 3 months postoperatively were analyzed, including demographic factors (gender, age), medical history (diabetes, hypertension), cardiac function status (BNP levels, LVEF), inflammatory markers (CRP levels), intraoperative parameters (surgery duration, cardiopulmonary bypass time), and postoperative complications. Univariate analysis revealed that patients with poor cardiac function improvement at 3 months postoperatively tended to be older, had a history of hypertension or diabetes, higher preoperative BNP levels, lower preoperative LVEF, and experienced more postoperative complications. Among these, age (*P* < 0.001), history of hypertension (*P* = 0.023), history of diabetes (*P* = 0.006), preoperative BNP levels (*P* = 0.012), preoperative LVEF (*P* = 0.025), and the number of postoperative complications (*P* = 0.036) were all significantly associated with poor cardiac function improvement at 3 months, identifying them as major risk factors affecting cardiac function recovery (Table 4).

**Table 4:** Univariate analysis of preoperative, perioperative, and postoperative variables associated with cardiac function improvement.

Variable	Cardiac function improved (*N* = 71)	Cardiac function not improved (*N* = 29)	*P*-value
Gender: male	42 (72.4%)	16 (16.8%)	0.714
Gender: female	29 (69%)	13 (31%)	
Age ≥63 years	31 (56.4%)	24 (43.6%)	**<0** **.** **001**
Age <63 years	40 (88.9%)	5 (11.1%)	
Hypertension	12 (52.2%)	11 (47.8%)	**0** **.** **023**
No hypertension	59 (76.6%)	18 (23.4%)	
Diabetes mellitus	5 (71%)	8 (29%)	**0** **.** **006**
No diabetes mellitus	66 (75.9%)	21 (24.1%)	
Preoperative BNP ≥1,363 pg/mL	27 (58.7%)	19 (41.3%)	**0** **.** **012**
Preoperative BNP <1,363 pg/mL	44 (81.5%)	10 (18.5%)	
Preoperative LVEF <36%	42 (80.8%)	10 (19.2%)	**0** **.** **025**
Preoperative LVEF ≥36%	29 (60.4%)	19 (39.6%)	
Preoperative CRP ≥3.84 mg/L	31 (63.3%)	18 (36.7%)	0.095
Preoperative CRP <3.84 mg/L	40 (78.4%)	11 (21.6%)	
Postoperative day 1 CRP ≥62 mg/L	34 (69.4%)	15 (30.6%)	0.728
Postoperative day 1 CRP <62 mg/L	37 (72.5%)	14 (27.5%)	
Postoperative 1 week BNP ≥752	40 (74. 1%)	14 (25.9%)	0.463
Postoperative 1 week BNP <752	31 (67.4%)	15 (32.6%)	
Postoperative 1 week LVEF ≥44%	35 (71.4%)	14 (28.6)	0.926
Postoperative 1 week LVEF <44%	36 (70.6%)	15 (29.4%)	
Surgery duration ≥278 min	34 (69.4%)	15 (30.6%)	0.728
Surgery duration <278 min	37 (72.5%)	14 (27.5%)	
CPB ≥128 min	33 (68.8%)	15 (31.3%)	0.634
CPB <128 min	38 (73. 1%)	14 (26.9%)	
Pulmonary infection	9 (56.3%)	7 (43.8%)	0.156
No pulmonary infection	62 (73.8%)	22 (26.2%)	
Renal dysfunction	5 (50%)	5 (50%)	0.123
No renal dysfunction	66 (73.3%)	24 (26. 1%)	
Arrhythmia	8 (57. 1%)	6 (42.9%)	0.218
No arrhythmia	63 (73.3%)	23 (26.7%)	
Postoperative complications ≤1	69 (73.4%)	25 (26.6%)	**0** **.** **036**
Postoperative complications ≥2	2 (33.3%)	4 (66.7%)	

Bold indicates that a *P*-value less than 0.05 indicates a statistically significant difference in the experimental results.

BNP, B-type natriuretic peptide; LVEF, left ventricular ejection fraction; CRP, C-reactive protein; CPB, cardiopulmonary bypass.

### Multivariate analysis comparison

Variables with statistical significance in the univariate analysis (age, hypertension, diabetes, preoperative LVEF, preoperative BNP levels, and the number of postoperative complications) were included in the binary logistic regression model for multivariate analysis. The results showed that older age significantly increased the risk of poor cardiac function recovery (OR = 5.833, 95% CI 1.793–19.014, *P* = 0.003), and lower preoperative LVEF was significantly associated with poorer cardiac function prognosis (OR = 5.962, 95% CI 1.472–24.123, *P* = 0.012) (Table 5).

**Table 5:** Multivariate logistic regression analysis of risk factors for cardiac function improvement.

Risk factor	*B*	SE	WaldX^2^	OR	*P*	95% CI for OR	
						Lower	Upper
Age	1.761	0.603	8.544	5.833	**0** **.** **003**	1.793	19.014
Hypertension	1.574	0.813	3.757	4.803	0.053	0.988	23.464
Diabetes mellitus	0.773	0.896	0.754	2.169	0.386	0.382	12.316
Preoperative BNP	0.785	0.552	2.001	0.461	0.157	0.165	1.357
Preoperative LVEF	1.792	0.715	6.275	5.962	**0** **.** **012**	1.472	24.123
Number of complications	0.750	1.092	0.484	2.123	0.491	0.253	17.870

Bold indicates that a *P*-value less than 0.05 indicates a statistically significant difference in the experimental results.

BNP, B-type natriuretic peptide; LVEF, left ventricular ejection fraction.

## Discussion

Valvular disease is one of the major causes of cardiac dysfunction and heart failure, significantly impairing both systolic and diastolic functions of the left ventricle as the disease progresses (17). Valve replacement surgery, as the primary treatment for moderate to severe valvular disease, can restore hemodynamic stability and significantly improve patient survival rates (18). However, although valve replacement effectively addresses mechanical dysfunction, postoperative inflammatory responses remain a major limiting factor for cardiac function recovery. These inflammatory responses may contribute to myocardial fibrosis and left ventricular remodeling, thereby impairing cardiac contractility and adversely affecting postoperative outcomes (19, 20). Therefore, early identification and effective management of postoperative inflammation are crucial for improving cardiac function in these patients.

Our study mainly found that LVEF in the high-inflammation group was significantly lower than that in the low-inflammation group at both 1 week postoperatively (43.55 ± 4.45 vs. 46.31 ± 4.84, *P* < 0.001) and 3 months postoperatively (50.96 ± 11.25 vs. 54.82 ± 6.52, *P* = 0.004). Meanwhile, BNP levels were significantly higher in the high-inflammation group at 1 week (751.63 ± 52.12 vs. 632.90 ± 305.10, *P* = 0.001) and 3 months (289.56 ± 108.51 vs. 186.99 ± 84.20, *P* < 0.001) postoperatively. This finding is consistent with the physiological role of BNP as a sensitive indicator of ventricular compensatory response, where elevated BNP levels typically indicate increased left ventricular wall stress and impaired cardiac function (21). Further analysis showed that the cardiac function improvement rate in the high-inflammation group (75.00%) was significantly lower than that in the low-inflammation group (80.68%), a result consistent with the findings of Patsalis et al. (13). These findings suggest that postoperative inflammation delays cardiac function recovery by increasing cardiac pressure load and inducing compensatory left ventricular dilation. The postoperative inflammatory response primarily originates from blood contact with foreign surfaces during cardiopulmonary bypass, ischemia-reperfusion injury of myocardial tissue, and the immune response triggered by surgical trauma (22, 23). During this process, inflammatory mediators such as C-reactive protein and interleukin-6 are continuously activated, leading to myocardial cell damage, restricted myocardial energy metabolism, and inhibition of functional recovery through the induction of myocardial fibrosis (13). After the activation of inflammatory mediators, the structural damage of myocardial cells is caused, such as the increase of cell membrane permeability and the leakage of cell contents, which leads to myocardial fibrosis. After the myocardial cells are damaged, energy metabolism is blocked, which may affect the recovery of myocardial function due to excessive protein decomposition or insufficient ATP production. Continued inflammation leads to the increase of apoptosis and the decrease of viable myocardial cells, further weakening myocardial function. In addition, inflammatory reaction triggers oxidative stress, affects mitochondrial function, leads to abnormal ATP production and aggravates energy metabolism problems. Additionally, our study reached a conclusion consistent with that of Keijmel (24), indicating that higher postoperative inflammatory responses significantly prolong hospitalization duration. Although there was no statistically significant difference in the incidence of postoperative complications between the two groups, the high-inflammation group experienced slower cardiac function recovery and persistent inflammatory states, leading to longer hospital stays than the low-inflammation group.

To further investigate the impact of preoperative and postoperative factors on cardiac function recovery after valve replacement surgery in the high-inflammation group, we conducted a univariate analysis of factors potentially influencing postoperative cardiac improvement. The analysis revealed that age, preoperative LVEF, preoperative BNP levels, history of hypertension and diabetes, and the number of postoperative complications were associated with poor cardiac function improvement.

Multivariate logistic regression analysis further demonstrated that age (OR = 5.833, 95% CI 1.793–19.014, *P* = 0.003) and preoperative LVEF (OR = 5.962, 95% CI 1.472–24.123, *P* = 0.012) were important independent risk factors affecting postoperative cardiac function recovery. In elderly patients, the number of myocardial cells decreases and the degree of myocardial fibrosis increases, resulting in the weakening of myocardial contractility. This physiological decline in myocardial function makes their cardiac reserve capacity relatively insufficient after such a major trauma as valve replacement surgery, which makes it difficult to effectively cope with the stress response caused by surgery, thus affecting the recovery process of cardiac function. Aging is accompanied by the gradual decline of immune system function, which is manifested by the decrease of immune cell activity and immune dysfunction. After valve replacement, the body will inevitably have a certain degree of inflammatory response, which is the normal response process of the body to surgical trauma. However, elderly patients have a poorly regulated immune system and are prone to excessive or persistent inflammatory responses. On the one hand, excessive inflammatory response will release a large number of inflammatory mediators, such as cytokines and chemokines, which can directly damage cardiomyocytes and destroy the normal structure and function of myocardium; on the other hand, the persistent chronic low-grade inflammatory state will inhibit the regeneration and repair ability of cardiomyocytes and hinder the recovery of cardiac function. In addition, low immune function may also lead to increased risk of infection, once infection occurs, it will further increase the burden of the heart, forming a vicious circle, seriously affecting the improvement of heart function. Myocardial inflammatory injury will lead to the decrease of myocardial cell survival rate and the decrease of myocardial contractility (leading to low LVEF). Conversely, low LVEF will promote the accumulation of inflammatory factors and further aggravate the myocardial inflammatory injury. These findings suggest that advanced age exacerbates postoperative recovery difficulties due to structural and functional deterioration of cardiac tissue. Meanwhile, patients with lower preoperative LVEF, experiencing prolonged left ventricular overload and limited compensatory capacity, have reduced potential for cardiac function recovery, consistent with the findings of Kolte and Sezai et al. (15, 25). Additionally, elevated preoperative BNP levels were significantly associated with poor cardiac recovery in the univariate analysis, suggesting that prolonged myocardial pressure overload may cause irreversible cardiac damage, impairing postoperative recovery. This aligns with the findings of Zhou et al. (26), who reported that elevated preoperative BNP levels typically reflect prolonged left ventricular pressure overload and an increased risk of underlying myocardial fibrosis. Although postoperative complications were significant in the univariate analysis, they did not emerge as independent risk factors in the multivariate regression analysis. This may be due to the combined effects of postoperative inflammation and complications, which could partially mask the independent effects of complications.

Postoperative inflammatory response is a key factor affecting cardiac function recovery. Previous studies have shown that optimizing cardiopulmonary bypass techniques and using anti-inflammatory drugs can effectively reduce inflammation levels and mitigate myocardial injury (27–29). Early monitoring of postoperative inflammatory markers is crucial for identifying high-risk patients. For instance, combined monitoring of CRP and BNP levels can help adjust anti-inflammatory treatment strategies and optimize cardiac function management, thereby improving cardiac recovery and shortening hospital stays (30). In the future, clinical research could further explore novel anti-inflammatory drugs or combination treatment strategies, particularly for high-risk patients with low preoperative LVEF and elevated BNP levels, to enhance postoperative management and improve long-term survival rates.

This study systematically explored the impact of preoperative and postoperative factors under inflammatory conditions on cardiac function improvement after valve replacement surgery. However, several limitations remain: (1) This was a single-center study with a relatively small sample size, which may limit the generalizability of the findings. Larger-scale, multicenter studies are required to validate these results. (2) The follow-up period was limited to 3 months postoperatively, preventing the assessment of long-term cardiac recovery and prognosis. Future studies should extend the follow-up duration to evaluate the incidence of chronic myocardial fibrosis and heart failure. And future research should be included in long-term follow-up (6 months, 1 year) to understand the progress of chronic myocardial fibrosis more deeply. (3) This study primarily used CRP as the postoperative inflammatory marker. Future research should include additional inflammatory markers, such as IL-6 and TNF-α, for a more comprehensive evaluation of inflammation's impact on cardiac function. Therefore, although this study preliminarily revealed the adverse effects of high inflammatory states on postoperative cardiac function, further validation through prospective, multicenter, long-term follow-up studies is necessary to optimize intervention strategies.

## Conclusion

The findings of this study demonstrate that elevated CRP levels on postoperative day 3 significantly affect cardiac function recovery after valve replacement surgery.

Patients in the high-inflammation group showed less improvement in LVEF, higher BNP levels, and longer hospital stays compared to the low-inflammation group.

Additionally, age and preoperative LVEF were identified as independent risk factors for postoperative cardiac function recovery. Therefore, comprehensive preoperative cardiac function assessments should be prioritized, particularly for older patients.

Based on these results, postoperative CRP levels can serve as an important early predictor of cardiac function recovery. This also underscores the importance of enhanced monitoring and intervention of inflammatory responses during perioperative management to potentially improve patient outcomes. Further prospective, multicenter studies with larger sample sizes are needed to validate these findings and explore more precise anti-inflammatory treatment strategies.

## Data Availability

The original contributions presented in the study are included in the article/Supplementary Material, further inquiries can be directed to the corresponding author.
